# Opportunities and Limits of Conventional IVF versus ICSI: It Is Time to Come off the Fence

**DOI:** 10.3390/jcm11195722

**Published:** 2022-09-27

**Authors:** Martina Balli, Anna Cecchele, Valerio Pisaturo, Sofia Makieva, Giorgia Carullo, Edgardo Somigliana, Alessio Paffoni, Paola Vigano’

**Affiliations:** 1 Infertility Unit, Fondazione IRCCS Ca’ Granda Ospedale Maggiore Policlinico, 20122 Milano, Italy; 2 Department of Clinical Sciences and Community Health, Università degli Studi di Milano, 20122 Milano, Italy; 3 Kinderwunschzentrum, Klinik für Reproduktions-Endokrinologie, Universitätsspital Zürich, 8091 Zurich, Switzerland; 4 Infertility Unit, ASST Lariana, 22063 Como, Italy

**Keywords:** infertility, IVF, reproduction

## Abstract

Conventional IVF (c-IVF) is one of the most practiced assisted reproductive technology (ART) approaches used worldwide. However, in the last years, the number of c-IVF procedures has dropped dramatically in favor of intracytoplasmic sperm injection (ICSI) in cases of non-male-related infertility. In this review, we have outlined advantages and disadvantages associated with c-IVF, highlighting the essential steps governing its success, its limitations, the methodology differences among laboratories and the technical progress. In addition, we have debated recent insights into fundamental questions, including indications regarding maternal age, decreased ovarian reserve, endometriosis, autoimmunity, single oocyte retrieval-cases as well as preimplantation genetic testing cycles. The “overuse” of ICSI procedures in several clinical situations of ART has been critically discussed. These insights will provide a framework for a better understanding of opportunities associated with human c-IVF and for best practice guidelines applicability in the reproductive medicine field.

## 1. Introduction

Infertility affects between 48.5 million couples, amounting to 186 million individuals worldwide [1]. Accordingly, assisted reproduction technology (ART) has widely become the most recommended practice for people who seek reproductive treatment. In nearly 40 years, remarkable progress has been made so that more than 40 years after the first test-tube baby, the International Committee for Monitoring ART (ICMART) reports that the global total of babies born as a result of ART procedures and other advanced fertility treatments is more than 8 million [2]. Moreover, advancements in controlled ovarian hyperstimulation, oocyte retrieval, embryonic culture conditions and scoring as well as in freezing procedures led to crucial accomplishments in human fertility treatments [3].

One of the most dramatic technological breakthroughs introduced in ART has been the intracytoplasmic sperm injection (ICSI), born to overcome the low and unpredictable fertilization rates encountered with conventional in vitro fertilization (c-IVF) in presence of poor sperm parameters [4]. However, the last two decades witnessed a rapid increase in the rate of ICSI use. This increase has been reported in several countries worldwide, with ICSI rate close to 100% in the Middle East. In the United States, ICSI use increased from 36% in 1996 to 76% in 2012 [5].

Conventional IVF and ICSI differ for the way eggs are fertilized: in the former, a woman’s eggs are surrounded by sperm in a Petri dish and ultimately one sperm fertilizes the egg, while in the latter, an embryologist selects a single sperm from a semen sample and injects it directly into the egg. Notably, the broadening of initial indications for ICSI over the years has not been substantiated by the improvement of ART outcomes [6]. In line, the National Institute for Health & Care Excellence (NICE) guidelines recommend the use of ICSI only for severe deficits of the sperm quality or for couples in whom a previous IVF treatment cycle has resulted in failed or very poor fertilization [7]. Based on the opinion of the Practice Committees of the American Society for Reproductive Medicine (ASRM) and the Society for Assisted Reproductive Technology (SART) on ICSI for non-male-factor indications, ICSI is also time- and resource-consuming compared to c-IVF [8]. Finally, the safety of ICSI remains of concern as it seems associated with an increased risk of congenital abnormalities and autism compared with c-IVF, although the underlying biological mechanisms are not known [9]. Overall, the expanded use of ICSI in couples with non-male-factor infertility shows a gap between clinical practice and evidence. Given the additional cost and invasive nature of this technique, the use of ICSI in ART should be always carefully appraised while c-IVF deserves critical attention from professionals involved in ART.

The purpose of this review was to evaluate the results of available publications dealing with the most controversial clinical and technical aspects of c-IVF. Methods and timing of the various steps as well as procedures to circumvent problems in c-IVF were investigated in depth. Special attention was paid to aspects for which systematic reviews or meta-analyses have not already defined the most scientifically correct strategy, with the aim to suggest refinements of the procedure and provide embryologists with a pragmatic tool for potential improvements of ART outcomes.

## 2. Conventional IVF: Technical Details

### 2.1. Timing of Insemination from Oocyte Retrieval

The debate regarding the effect of the timing of insemination after oocyte retrieval started in the 1980s, when three different groups demonstrated better fertilization rates and higher embryo quality when oocytes were inseminated after a pre-incubation period of 3–5.5 h after retrieval [10,11,12,13]. In the same years, the group of Fish and coworkers showed that different pre-insemination intervals (within 9 h after oocyte retrieval) did not affect fertilization or pregnancy rates [13]. More recent studies focused on the impact of the pre-incubation period on the fertilization rate were similarly not concordant. Ho et al. reported significantly better results by performing the insemination in a time window ranging from 2.5 to 5.5 h after oocytes retrieval. Specifically, they analyzed the fertilization rate at <2.5 h, <3.5 h, <4.5 h, ≤5.5 h and >5.5 h after oocyte retrieval and observed fertilization rates of 67.9%, 80.5%, 82.0%, 84.5% and 73.0%, respectively. They obtained statistically significant data when the insemination was performed <2.5 h and 5.5 h after oocyte retrieval (*p* < 0.001) [14]. In contrast, Jacobs and colleagues demonstrated different results analyzing the fertilization rate, embryo quality, implantation rate, abortion and ongoing pregnancy when inseminations were performed 1–7 h after oocyte retrieval. No statistically significant differences in c-IVF outcomes performed in different time intervals were observed, suggesting that early insemination could be performed without reservation [15]. Esiso and colleagues divided the time interval between oocyte retrieval and insemination into eight categories: 0 (0–<0.5 h), 1 (0.5–<1.5 h), 2 (1.5–<2.5 h), 3 (2.5–<3.5 h), 4 (3.5–<4.5 h), 5 (4.5–<5.5 h), 6 (5.5–<6.5 h) and 7 (6.5–<8 h). The relative number of oocytes retrieved in each category was *n* = 586, *n* = 1594, *n* = 1644, *n* = 1796, *n* = 1836, *n* = 1351, *n* = 641 and *n* = 127, respectively. Considering only the c-IVF outcome, they had optimal results when the insemination was performed between 1.5 h and 6 h after oocyte retrieval. When performed prior to 1.5 h, the detrimental effects of insemination on the fertilization rate were only moderate, without affecting blastulation and pregnancy rates [16]. Overall, these studies demonstrate the existence of an optimum time range for more successful c-IVF that should be performed between 3 and 6 h after oocyte retrieval. Of note, the time elapsed from ovulation trigger and oocyte retrieval (generally 34–36 h) should be taken into account when evaluating the proper timing of insemination.

### 2.2. Timing of Sperm-Cumulus Co-Incubation

The standard human c-IVF method involves overnight insemination of cumulus-intact oocytes with a defined range of spermatozoa/mL, followed by a fertilization assessment in the next morning [17]. It has been, however, suggested that extended gametes co-incubation may lead to unsuccessful c-IVF due to the production of high concentrations of reactive oxygen species (ROS), potentially deleterious for oocyte and embryo quality [18,19,20,21]. Therefore, to prevent unfavorable effects on oocytes and related embryos from high ROS exposure, a shorter incubation of gametes has been proposed. Studies addressing the comparison between short gametes co-incubation to standard overnight IVF (16–18 h) are listed in Table 1 [22,23,24,25,26,27,28,29,30,31,32,33,34,35,36,37,38,39,40,41]. Yet in 1996, Gianaroli et al. reported that a short gamete co-incubation (1 h) led to an increased rate of oocyte fertilization and embryo viability, suggesting that prolonged exposure of oocytes to elevated concentrations of sperm cells may negatively influence early embryo development [22]. In accordance, Le Bras and colleagues demonstrated that short gamete co-incubation period (2 h) leads to higher embryo quality, with a percentage of fragmentation lower than 25%, and, most importantly, to a significant increase in clinical pregnancy after a fresh embryo transfer compared to standard overnight insemination (16–18 h) [23]. Other studies reported that a shorter oocyte-spermatozoa incubation time is associated with an enhanced embryo quality and could prevent total fertilization failure (TTF) [24,25]. Conversely, in a prospective study, Barraud-Lange et al. evaluated the effects of a short gamete co-incubation (1 h) on fertilization rate and embryo quality of sibling oocytes, showing a decreased fertilization rate and comparable embryo quality compared to the standard overnight insemination method [26]. The meta-analysis from Zhang et al., published in 2013, revealed that reduced gamete co-incubation time is associated with beneficial outcomes, including significantly increased clinical pregnancy rate (Pooled Risk Ratio [RR]: 1.84, 95% confidence interval (CI) 1.24–2.73), ongoing pregnancy rate (RR: 1.73, 95% CI: 1.27–2.33) and implantation rate (RR: 1.80, 95% CI: 1.43–2.26) compared to c-IVF, with no significant differences in fertilization rates, embryo quality and polyspermy rate (RR: 0.98, 95% CI: 0.93–1.02; RR: 1.24, 95% CI: 1.0–1.53; RR: 0.84, 95% CI: 0.7–1.01, respectively) [27]. A recent randomized study of *n* = 320 infertile women, evaluated the beneficial influence of short gamete co-incubation in terms of live birth rate. Here, oocytes of patients randomized to the short co-incubation period group (3–4 h, *n* = 160) and to the conventional co-incubation timing (20 h, *n* = 160) were inseminated with ~20,000–30,000 motile spermatozoa/oocyte. Contrarily to other studies, no statistically significant difference in terms of live birth rate, clinical pregnancy, miscarriage and implantation rates was reported between the short-time and standard overnight insemination groups [28]. Overall, evidence on which is the best timing of sperm-egg co-incubation in c-IVF requires more consolidation.

### 2.3. Oxygen Tension

Embryo development depends on a variety of factors, and among others, oxygen tension represents one of the key decisive parameters to ensure proper embryogenesis [28]. Mammalian development is characterized by low concentration of oxygen in both fallopian tubes and uterus, ranging from 5 to 7% in the former and decreasing to 2% in the latter [25]. Therefore, in order to support proper embryo development, it may be crucial to mimic the low level of oxygen tension (5%) present in the in vivo developmental environment. Interestingly, several studies reported that successful human embryo culture, in terms of embryo quality and blastulation rate, occurs under hypoxic conditions (5% oxygen tension). Moreover, the rate of implantation, pregnancy, good-quality embryos for transfer and live birth significantly increases under hypoxia culture conditions rather than with atmospheric oxygen levels [42,43,44,45,46]. The oxidative stress resulting from high oxygen concentration culture conditions may severely affect embryo quality, decreasing its implantation potential and, as a consequence, the pregnancy rate [43,45,47]. Moreover, embryos exposed to ROS are prone to DNA damage and mitochondrial alterations, leading to activation of the apoptotic mechanism [48]. Data obtained from a meta-analysis showed a notable improvement on live birth rate following embryo culture in low oxygen concentration. The rate of live births was improved up to 43% by lowering the oxygen concentration during embryo development [49]. Accordingly, recommendations provided from the latest ESHRE guidelines suggest the use of low oxygen concentration for embryo culture [50]. Nonetheless, the oxygen tension used in the culture system has ample differences among ART laboratories worldwide [51]. Notably, the impact of oxygen tension on results of ICSI versus c-IVF in relation to stage-specific sensitivity is unclear. Most of the papers on this topic do not divide the results according to the two strategies. In a prospective randomized sibling-oocyte study, Guo et al. evaluated the impact of different oxygen concentrations (20% versus 5%) on fertilization rates in c-IVF cycles. A total of *n* = 1254 oocytes were randomly assigned to 20% or 5% oxygen tension culture conditions on the retrieval day and then treated with c-IVF. The two groups did not show differences in fertilization rate, suggesting that at this stage of development, different oxygen tensions may not influence the process of fertilization. However, oocytes cultured at 5% oxygen gave rise to an increased number of optimal embryos on day 3 (72.4% vs. 64.2%, respectively, *p* = 0.018) and higher blastocyst formation rate (64.5% vs. 52.9%, respectively, *p* = 0.009) compared to the 20% group. Moreover, the use of low oxygen tension resulted in a more favorable clinical pregnancy and implantation rates compared with atmospheric oxygen [52]. In a total of *n* = 402 ART cycles, Guarneri et al. specifically evaluated the use of two different oxygen concentrations (atmospheric versus low oxygen) during oocyte culture from recovery until decumulation on day 1, followed by use of low oxygen concentration (5%) until transfer. Interestingly, cumulus-intact oocytes cultured in atmospheric oxygen tension for ~20 h for c-IVF resulted in a comparable number of transferred/vitrified embryos from the inseminated oocytes, cumulative clinical pregnancy rate and cumulative live birth rate per cycle compared to the oocytes cultured under 5% oxygen level from gamete retrieval to embryo transfer [53]. Although evidence-based data strongly indicate that culturing embryos in low oxygen concentration improves embryo utilization rate and increases the chance of pregnancy [50,54], the potential antioxidant activity of the cumulus cells present during the first step of c-IVF needs to be further investigated.

## 3. Conventional IVF, ART Indications and Clinical Situations

### 3.1. Should We Use Conventional IVF for an Indication of Advanced Maternal Age?

According to Centers for Disease Control and Prevention (CDC) ART reports [55], in the United States, the percentage of fresh non-donor cycles in patients with non-male-factor infertility in which ICSI was used increased with increasing female age at least until 2015, when it was 69% in younger women (<35 years) and 80% in women > 44 years of age. Although the increasing use of ICSI for non-male-factor infertility cases has been shown not to improve live birth rates [8], the advanced maternal age still represents a possible indication for ICSI in several clinics. The underlying rationale is based on the idea that oocytes from advanced-age women have functional or structural defects that might interfere with the fertilization process. On the other hand, a recent meta-analysis of observational studies strongly suggests that their fertilization rate is similar using both c-IVF or ICSI [56]. Seven studies from 2001 to 2016 were included in the meta-analysis and the pooled RR for fertilization in ICSI compared to c-IVF cycles was 0.99 (95%CI: 0.93–1.06), indicating no significant difference in fertilization rates between the two techniques in women > 38 years with normozoospermic partners. A randomized in vitro clinical trial with sibling oocytes allocated to c-IVF or ICSI showed that ICSI does not improve the reproductive outcomes of advanced-age patients undergoing conventional insemination for non-male-factor infertility in terms of fertilization rate and embryo development [57].

Even considering pregnancy/live birth rates according to the insemination technique in advanced maternal age women, most available data fail to demonstrate an advantage of ICSI over c-IVF. According to CDC ART reports, c-IVF is generally more efficient in all women age groups in the absence of a male factor of infertility. A similar trend can be extrapolated from studies included in the previously mentioned meta-analysis [56]. When available data were pooled together, pregnancy rate per cycle was 13.1% (*n* = 127/967) in c-IVF and 7.3% (*n* = 61/831) in ICSI cycles (*p* < 0.001). Live birth rates per c-IVF and ICSI cycles in women aged 40 years or more were 12.2% versus 6.0% in Liu et al. [58] and 18.8% versus 14.5% in Tannus et al. [59], respectively. Pooled live birth rates per cycle in women aged 40 years or more were 9.6% (76/789) and 7.7% (46/600) in c-IVF and ICSI, respectively (*p* = 0.20).

At present, ICSI does not seem to improve fertilization rate or live birth outcomes for an indication of advanced maternal age.

### 3.2. Should We Use Conventional IVF for an Indication of Decreased Ovarian Reserve?

The use of ICSI has also been proposed as a strategy to manage cases of poor ovarian reserve (POR), diminished ovarian reserve (DOR) and low oocyte yield in the absence of male factor infertility. We identified 10 studies that examined whether ICSI improves embryological and/or clinical outcomes in women with limited number of oocytes available for treatment after controlled ovarian hyperstimulation in the absence of male factor infertility (Table 2). Despite the fact that eight of these studies were conducted after the publication of Bologna criteria in 2014 [60], they did not all define their study group accordingly, with some considering an arbitrary low number of oocytes as an indication of POR. Only one study has been conducted in strict accordance with the Bologna fulfillment criteria. That was a retrospective analysis comparing c-IVF (*n* = 72) and ICSI (*n* = 164) cycles of women ≥ 40 years of age who had ≤3 oocytes available for treatment in the absence of male factor infertility [59]. Fertilization rate, clinical pregnancy rate and live birth rate were all statistically comparable between the two groups, with c-IVF achieving 7.8% and ICSI 4.3% live birth rates. The rate of fertilization failure, albeit on the higher end, was also similar after c-IVF (26.3%) and ICSI (22.5%). Only three studies considered ≤3 oocytes as the sole indication of a poor ovarian reserve. The largest of them, a Human Fertilisation and Embryology Authority (HFEA) registry analysis of cycles performed between 1998–2016, demonstrated that c-IVF (*n* = 33,436) and ICSI (*n* = 29,205) result in similar clinical pregnancy (~14%) and live birth rate (~12%) when adjusted for female age and previous ART attempts [61]. However, fertilization rate was 2% higher in the ICSI group, while failed fertilization rate was comparable. Nonetheless, the authors argued that this result should be interpreted as showing no evident benefit of ICSI over c-IVF in the presence of normal sperm parameters. The most recent retrospective analysis of women with ≤3 oocytes available also concluded that low oocyte number is not an indication to perform ICSI when the sperm parameters are within normal WHO ranges and the decision of the insemination method should be solely based on semen quality [62]. Indeed, implantation rate, live birth rate and fertilization failure rate were similar when the cycles of *n* = 77 c-IVF and *n* = 65 ICSI patients were compared. In contrast to the HFEA study, the authors found a 13.1% higher fertilization rate per collected oocyte in the c-IVF group. Despite the low number of patients, the study holds strong credibility due to the inclusion of an additional group accounting for the statistics, an ICSI group in presence of male factor infertility.

Three of the 10 studies identified (Table 2) examined the gold standard of ART outcomes, the cumulative live birth rate. The first study, a multicenter retrospective analysis, found comparable cumulative live birth between women aged 34–40 years treated with c-IVF (*n* = 90) or ICSI (*n* = 600) with 1–3 oocytes retrieved in 14 European centers between 2009 and 2014 [63]. Notably, the fertilization rate was identical in the two groups. The second study similarly reported cumulative live birth to be independent of the fertilization technique in a retrospective analysis of cycles from *n* = 870 c-IVF-and *n* = 435 ICSI-receiving women who yielded 1, 2, 3 or 4 oocytes [64]. The third study was the only to argue the advantage of c-IVF over ICSI in first cycles of women aged ≥40 years with ≤5 oocytes retrieved and non-male-factor infertility [58]. Specifically, they found higher cumulative live birth in women who received c-IVF, although the fertilization rate was lower. Two more retrospective studies, adopting a less stringent definition of what is a low oocyte yield, namely ≤5 or 6 oocytes, report similar outcomes between c-IVF and ICSI in cycles with normal sperm parameters, with one of them even reporting no differences in perinatal outcomes [65,66]. Only two prospective studies have been performed comparing c-IVF and ICSI outcomes in normozoospermic cases. Moreno and colleagues (1998) randomized sub-fertile women with ≤6 retrieved oocytes to either c-IVF (*n* = 52) or ICSI (*n* = 52) and noted no differences in embryo quality, fertilization rate, fertilization failure, implantation rate and clinical pregnancy rate [67]. Interestingly, live birth was not reported in this study and the cohort was recruited between 1996 and 1997; hence the findings might not be applicable to current practices. The second prospective study was designed to recruit women of advanced maternal age, not POR, but the cohort could still be considered in the context of POR due to the average number of oocytes per group, which was 4.3 [57]. As mentioned above, that study randomized *n* = 258 and *n* = 257 sibling oocytes to c-IVF and ICSI, respectively, and found similar fertilization rate, top-quality embryo rate and cleavage rate between the two groups. Due to the design of the study utilizing sibling oocytes, the live birth rate was not assessed. Overall, all the aforementioned studies highlight that the choice of insemination method should not be based on the number of oocytes retrieved and routine attribution of ICSI to poor responders is not justified on the sole basis of their oocyte yield. The fertilization rate, which appears significantly different between c-IVF and ICSI in some studies, should be interpreted with caution as the denominator of this outcome varies, with some studies considering the number of collected cumulus-oocyte complexes and others the number of inseminated oocytes. In any case, none of the studies could detect a significant difference in the rate of fertilization failure or cancellation rate of embryo transfer, which is an argument often placed on table when defending the ICSI approach for poor reserve patients. A randomized controlled trial powered to answer whether ICSI is superior in management of women who fall under the POR definition according to Bologna criteria in the absence of male factor infertility is yet to be conceptualized.

### 3.3. Should We Use Conventional IVF for an Indication of Endometriosis?

The rationale to use ICSI for endometriosis patients is based on the supposed reduction of fertilization rate in these patients. Oocytes retrieved from women affected by endometriosis have been suggested to more likely fail in vitro maturation, to show altered morphology and to have lower cytoplasmic mitochondrial content [68]. Similarly, in the oocytes of women with advanced maternal age, these alterations are thought to interfere with the fertilization process. Surprisingly, a single study was set up in order to compare c-IVF or ICSI to fertilize oocytes from couples with endometriosis and normozoospermic semen [69]. Sibling oocytes (*n* = 786) were randomized to be inseminated by the same semen sample either with c-IVF (*n* = 387) or ICSI (*n* = 399). The fertilization rate resulted significantly higher in the ICSI group compared with the group inseminated by c-IVF (73.3 ± 23% versus 54.7 ± 31.9%, respectively, *p* = 0.003). The best embryos were then selected for transfer independently of their mode of insemination, preferably involving only one type of insemination. There were no statistically significant differences in implantation, pregnancy, chemical pregnancy, clinical abortion and ongoing pregnancy rates between the two groups. More and larger studies are needed to understand whether ICSI can provide better outcomes than c-IVF in these patients.

### 3.4. Should We Use Conventional IVF in Couples with Autoimmunity?

Autoimmune diseases affect couples of childbearing ages and may negatively impact reproductive health [70]. Sperm cells exhibit antigens extraneous to both male and female immune systems. With the exposure of these antigens to immune cells, antisperm antibodies (ASAs) are naturally formed. ASAs have been detected in men with infertility and women with unexplained infertility [71]. Antibodies in the semen may negatively interfere with the fertilization process through various mechanisms, such as inhibition of sperm motility and sperm progression through the female genital tract, altered acrosomal reactions, or binding to zona pellucida [72]. In particular, ASA interfering with the sperm penetration into the zona pellucida may have a consistent role in the fertilization failure following c-IVF. Studies on the fertilization rate following c-IVF reported controversial data. Although some authors showed a detrimental effect of antisperm antibodies on fertilization rate [73,74], others found no significant differences between couples with ASA-positive and ASA-negative male partners, demonstrating that the presence of ASA may not affect fertilization rate and clinical outcomes following c-IVF [75,76]. Lu and coworkers investigated the fertilization rates of infertile couples with a serum ASA-positive or ASA-negative partner who underwent c-IVF or ICSI cycles. In c-IVF cycles, a decrease in fertilization rate was observed in couples with ASA-positive male partners compared to ASA-negative male partners (41.7% ± 23.4% vs. 54.8% ± 29.9%, *p* = 0.03, respectively). In addition, significantly lower clinical pregnancy and live birth rates were observed in the ASA-positive group. Conversely, no differences were found in terms of fertilization, pregnancy and live birth rates in couples with ASA-positive male partners compared to ASA-negative male partners treated by ICSI, suggesting that ICSI might overcome issues derived from ASA [77]. In this context, ICSI has become an alternative for managing couples affected by ASAs. Microinjection of a sperm into the oocyte can minimize the negative effects of ASAs on binding between spermatozoa and zona pellucida and other subsequent events of fertilization. Some studies have consistently shown that ASAs do not affect fertilization and pregnancy rates following ICSI [78,79] (Table 3). It is important to note that all these studies are quite dated, raising doubts about the relevance of the antisperm antibodies as a mechanism interfering with fertility.

Among immunity condition affected women, thyroid autoimmunity presents a greater prevalence in infertile women than fertile subjects [80]. Serum antibodies directed against thyroperoxidase expressed in thyrocytes or thyroglobulin produced by the thyroid gland represent markers of this disorder. Monteleone et al. [81] hypothesized that these anti-thyroid antibodies might bind antigens expressed in the zona pellucida, resulting in detrimental effects on fertilization rate and embryo quality. Comparing patients with thyroid autoimmunity with negative controls undergoing ART cycles, they reported a reduction in terms of embryo quality, fertilization and pregnancy rates. In line with this hypothesis, the authors suggested that the use of ICSI would overcome the negative impact of thyroid autoantibodies. Other studies analyzed the effect of thyroid autoimmunity on fertilization rate [82,83,84]. However, in all studies that evaluated this effect, ICSI was performed. Therefore, the lack of data from women undergoing c-IVF cycles prevents any firm conclusions [85]. Although different studies reported negative effects of this condition on clinical outcomes, no data have been reported demonstrating the superiority of ICSI over c-IVF in couples with autoimmunity.

### 3.5. Should We Use Conventional IVF in PGT Cycles?

Preimplantation genetic testing (PGT) is used for the identification of abnormal embryos carrying various forms of genetic abnormalities in order to allow the transfer of genetically healthy embryos [86,87]. PGT is offered to couples with a high risk of monogenic disorders (PGT-M) or chromosomal structural rearrangements (PGT-SR) or for the identification of chromosomal aberrations or aneuploidies (PGT-A) [88]. Microinjection is specifically recommended by ASRM, SART, the European Society of Human Reproduction and Embryology and the PGD International Society (ESHRE/PGDIS) in PGT cycles, regardless of the semen parameters [89,90,91,92]. The motivation to choose ICSI is to minimize any paternal contamination represented by spermatozoa attached to the zona pellucida. Moreover, the denudation of oocytes from the cumulus cells prior to the microinjection prevents any maternal contamination, although the enzymatic treatment combined with mechanical removal is not always successful in removing all cumulus cells before ICSI [93]. Several published works addressed the issues on whether ICSI is actually the best choice in case of PGT, in particular in terms of safety to avoid extraneous sperm DNA contamination (Table 4). Feldman and colleagues investigated this aspect by performing a cohort-historical study of all consecutive patients characterized by non-male infertility admitted to the IVF-PGT-M program in their center. Nine hundred and twenty-seven cycles were included in the study and were divided into three groups: a c-IVF group counting *n* = 315 cycles (where all the oocytes underwent c-IVF only), an ICSI group counting *n* = 565 cycles (where all oocytes underwent ICSI) and a mixed group counting *n* = 47 cycles (where sibling oocytes underwent both c-IVF and ICSI). They obtained comparable results between oocytes undergoing c-IVF and ICSI in terms of fertilization rate and percentages of embryos undergoing biopsy per fertilized oocyte. Comparable percentages of embryos with a complete diagnosis and comparable percentages of unaffected/transferable embryos were obtained. No significant difference in the percentage of paternal contribution to abnormal embryos and no significant difference in contamination rates of the washing medium samples after c-IVF or ICSI were reported. Importantly, their overall outcomes of c-IVF and ICSI cycles were comparable to the ESHRE consortium data collection XIII [94]. With this study, the authors demonstrated that ICSI is not superior to c-IVF in ensuring a lower risk of contamination from extraneous sperm attached to the zona pellucida or non-decondensed sperm within blastomeres or cumulus cells [95]. Other published studies addressing the same question demonstrated that performing c-IVF or ICSI in PGT-M cycles or FISH leads to the same euploid rates [96,97] (Table 4). Palmerola and colleagues and in parallel the group of De Munck assessed the accuracy of c-IVF versus ICSI in PGT-A cycles by looking at the difference in the prevalence of aneuploidy and mosaicisms [93,95,97,98]. In general, they found no association between c-IVF and higher prevalence of aneuploidy or mosaic embryos. On the other hand, Palmerola et al. reported a trend toward a higher rate of mosaicisms after conventional insemination. This result could be due to biological mechanisms (i.e., the effect of the insemination methods on embryo development and on chromosome segregation during subsequent mitotic divisions) or to the technical artifact related to genetic contamination. Anyway, their analysis did not achieve statistical significance. They also confirmed that c-IVF yields comparable results to ICSI in terms of number of euploid embryos per oocyte. Taken together, these observations demonstrate that ICSI does not ensure a lower exogenous DNA contamination compared to c-IVF, which could be used in all the PGT cycles characterized by non-male-factor infertility (Table 4).

### 3.6. Should We Use Conventional IVF in Single Oocyte Retrievals?

ICSI is often used for non-male-factor infertility based on the assumption that this technique might avoid unexpected TFF. In line with this principle, ICSI should be recommended in case of women with a single oocyte retrieved. Different studies have assessed the effectiveness of c-IVF in cases where only one oocyte is available for insemination, contradicting this theory. Gozlan and colleagues compared the efficacy of ICSI and c-IVF in patients with normal and subnormal sperm parameters in which only a single oocyte was available for insemination [99]. On *n* = 209 single oocytes inseminated with normal semen, no statistical significance was observed comparing ICSI versus c-IVF in terms of fertilization rate (female age < 39 years, 75% vs. 67.1%, respectively; female age > 39 years, 82.4% vs. 68.4% respectively). On the other hand, as expected, in *n* = 209 oocytes inseminated with subnormal semen, ICSI proved to be more efficient than c-IVF in terms of fertilization rate (female age < 39 years, 85.4% vs. 44.2%, respectively, *p* = 0.0001; female age > 39, 84.0% vs. 52%, respectively, *p* = 0.0003). In line with these results, Luna et al. evaluated *n* = 350 ART cycles in which four or fewer oocytes were retrieved, finding similar fertilization rates between c-IVF and ICSI per oocyte retrieved (51.5% vs. 51.8%, respectively) [65]. However, in this study, the number of cases with a single oocyte was very low (*n* = 7 with c-IVF and *n* = 4 with ICSI). Additionally, Sfontouris et al., comparing c-IVF and ICSI on *n* = 243 cases with a single oocyte retrieved, found similar fertilization rates (65.3% with c-IVF and 66% with ICSI) [100]. No differences were also found in terms of live birth rates per oocyte retrieval (5.0% with c-IVF and 4.0% with ICSI) and implantation rates (8.9% with c- IVF and 10% with ICSI) between the two techniques. All the studies considered are limited by their retrospective nature. Nevertheless, based on the available data, for cases of non-male-factor infertility, the use of c-IVF is not contraindicated when a single oocyte is retrieved. Randomized controlled trials are needed to definitively clarify this issue.

## 4. Conventional IVF and Male Gametes

### 4.1. What Characteristics of Spermatozoa in the Ejaculate to Consider Conventional IVF?

Sperm quality is considered decisive to ensure favorable c-IVF results [101]. In a retrospective study from Villani et al., sperm motility positively predicted the occurrence of fertilization (statistical accuracy = 71.1%), pregnancy and live birth rates in couples treated with c-IVF [102]. Moreover, the total number of spermatozoa in the ejaculate and the concentration of cells are strictly related to pregnancy rates and, therefore, considered predictors of conception [103]. According to WHO2021, a concentration of ≥39 × 10^6^/mL of spermatozoa cells in the ejaculate, with a progressive motility of 30% and 4% of normal morphology, may be considered reference limit values to allow appropriate oocyte fertilization [104]. In a recent randomized trial performed by Dang et al., couples with non-male-factor infertility were treated with c-IVF (*n* = 532) or ICSI (*n* = 532). Although some ART outcomes appeared to be improved following ICSI treatment, both the rate of live birth after embryo transfer and ongoing pregnancies did not significantly differ between the two groups. A greater level of fertilization was indeed obtained upon ICSI (75%) treatment compared to the c-IVF group (66.7%; *p* < 0.0001). Of note, sperm morphology was not evaluated on the day of c-IVF, and in the diagnostic phase, the median (IQR) percentage of normal sperm cells was 3% (1–6%); this value configured a sample of males, which, despite having counts and motility similar to those of the fertile population, showed as much as 60% of subjects with sperm morphology below the 5th percentile. This observation can mask important indications on the generalizability of the results [105].

The decision of the best treatment option is more difficult in case of moderate oligozoospermia or oligoasthenozoospermia [106]. Shuai and colleagues demonstrated that moderate oligoasthenozoospermia does not negatively affect c-IVF clinical outcomes, such as fertilization, implantation and pregnancy rates, compared to ICSI treatment. Moderate oligozoospermia was defined as having no history of any male accessory gland infection, a sperm count of 5 × 10^6^ sperm/mL to 20 × 10^6^ sperm/mL and a progressive motility <32%. However, the number of top-quality embryos appeared to be greater following ICSI treatment [107]. In a randomized study from van der Westerlaken et al., *n* = 1518 sibling oocytes were randomly inseminated via ICSI or c-IVF with borderline semen samples in order to determine the optimal treatment choice. Borderline semen samples were defined by the presence of at least one abnormal semen parameter, such as a sperm concentration lower than 20 × 10^6^/mL and/or reduced sperm motility (<40%). Here, the ICSI-inseminated oocytes resulted in higher fertilization rates per oocyte compared to c-IVF (50% vs. 41%, respectively). Moreover, the use of c-IVF in cases of sub-optimal semen samples led to a greater risk of TFF (24%) compared to ICSI-inseminated oocytes (1.8%). However, once fertilization was established, no notable differences were obtained in terms of pregnancy rates and ongoing pregnancies between the groups [108]. The impact of moderate male infertility on fertilization capacity was also assessed in the work of Xie et al., where *n* = 249 couples with moderate male infertility were treated. Sibling oocytes were randomized into groups to be inseminated either by c-IVF or ICSI. Male inclusion criteria were sperm count 5–20 × 10^6^/mL and/or sperm with progressive motility >10–32% and no infection of accessory glands. Fertilization rate (74.4% vs. 72.1%), implantation (36.3% vs. 30.1%) and pregnancy rate (57.9% vs. 43.3%) did not differ between c-IVF- and ICSI-inseminated oocytes in the oligozoospermic group. Interestingly, the percentage of top-quality embryos significantly increased after ICSI compared to c-IVF (33.6% vs. 24.5%) [106]. Outcomes have also been suggested to be negatively influenced by sperm morphological abnormalities. Particularly, severe teratozoospermia, indicated with normal sperm morphology ≤4%, represents one the causes of potential decreased fertilization or TFF in patients undergoing c-IVF [109,110]. Although specific and rare conditions such as globozoospermia can be incompatible with fertilization in c-IVF, findings are discordant regarding the choice of the insemination technique with ordinary cases of teratozoospermia. Accordingly, Zhu and colleagues analyzed c-IVF outcomes in couples affected by severe defective sperm morphology (>98%). Conventional IVF-derived embryos displayed a notable reduction in cleavage rate, biochemical/clinical pregnancy rate, live birth rate and miscarriage rate compared to the control group [111]. In contrast, in a retrospective study by Keegan and colleagues, no significant differences were observed in fertilization, pregnancy and live birth rates after performing c-IVF in couples with isolated teratozoospermia (<5% normal sperm morphology) compared to the control group (≥5% normal sperm morphology) [112]. Similarly, Fan and colleagues observed that fertilization, good-quality embryo, implantation, clinical pregnancy and miscarriage rates were not different when treated with c-IVF or ICSI based on the presence of isolated teratozoospermia [113]. A recent publication from Stimpfel et al. reported a significant beneficial effect of c-IVF in the treatment of couples whose infertility was attributed to teratozoospermia (defined by less than 15% of normal spermatozoa). Sibling oocytes from *n* = 51 couples were treated with both c-IVF and ICSI. A reduced number of mature oocytes were degenerated following c-IVF compared to ICSI (4.3% vs. 11.7%; *p* = 0.0003), and an increased number of top-quality blastocysts was obtained (29.2% vs. 19.8%; *p* = 0.037). Fertilization rate, embryo quality and pregnancy rate displayed similar results, although it was observed a positive trend in favor of c-IVF in terms of pregnancy [114]. Therefore, given the highly controversial findings, it is evident that in presence of conditions of borderline semen parameters in terms of number, motility and morphology, the decisions on the strategy of fertilization is, even today, completely dependent on the politics, results and attitude of the specific embryology laboratory.

Finally, although studies have reported that polyzoospermia, which is caused by elevated spermatozoa concentration (~250 × 10^6^ cell/mL), may lead to improper fertility and high miscarriage rates, the nature of the defect is controversial [115]. Therefore, maximum reference values for sperm concentration are considered clinically irrelevant, since we lack proof demonstrating that elevated sperm concentration represents a burden to fertility [116]. Overall, nowadays more informative randomized studies and protocols are strictly required in order to gain more evidence on whether ICSI or c-IVF is the most suitable ART treatment method in couples affected by non-severe male infertility [117].

### 4.2. Which Features Should Spermatozoa Possess to Ensure Successful Conventional IVF?

As previously mentioned, appropriate progressive motility is one of the key sperm features to allow both natural and assisted conception [118,119,120,121]. Total motile sperm count is widely adopted for c-IVF eligibility. Low total motile count has been reported to be associated with abnormal cell number in day 3 embryos and as well as with poor day 3 cell symmetry [122]. Usually, post-treatment, 2 to 5 million motile spermatozoa/mL are recommended for c-IVF. However, the number of spermatozoa used for insemination of cumulus-intact oocytes during c-IVF differs between laboratories, ranging from 2 × 10^4^ to 5 × 10^4^ post-treatment motile spermatozoa in a final volume ranging from 20 μL to 1 mL [123,124,125]. Specific sperm kinematic factors are also taken into consideration when performing c-IVF. Among those, sperm curvilinear velocity (VCL), straight-line velocity (VSL), average path velocity (VAP) and amplitude of lateral head displacement (ALH), measured using computer-assisted analysis, have been shown to be efficiently able to anticipate c-IVF outcome [126]. VSL and VCL could be implemented as a prognostic value in order to predict the fertilization potential of the semen sample. Specifically, with a sample characterized by a VSL > 40 μm/s, c-IVF should be considered [121]. In addition, sperm cells can be classified based on the speed at which the cell moves with flagellar movement. Rapid sperm cells display a progressive motility > 25 μm/s (micrometers per second) and can mainly swim in a straight line. On the other hand, slow progressive sperm cells move much more slowly and usually not in a straight line; these cells exhibit a speed of <25 μm/s. Non-progressive motility indicates sperm cells characterized by a speed < 5 μm/s [127]. Consequently, in order to be considered “healthy”, a semen sample should display ≥50% of both rapid and slow progressive sperm cells. However, the impact of post-treatment sperm morphology on c-IVF success remains to be clarified.

### 4.3. How to Prepare the Semen?

Different sperm preparation methods for c-IVF have been described over time in the literature. They were all developed with the purpose of maximizing the concentration of good-quality sperm cells and eliminating seminal plasma, debris and other harmful substances that could have negative effects (i.e., bacterial contamination or compounds causing uterine contractions). All the techniques are aimed to reduce immotile sperm, immature sperm cells and leukocytes from the final volume to be inseminated in order to obtain the higher sperm percentage with normal morphology and progressive motility [128]. Moreover, the ideal sperm preparation methods should ensure the preservation of sperm physiological conditions, avoiding irreversible injuries in sperm membranes and sperm DNA [129,130]. The most commonly used include density gradient centrifugation (DGC) and swim-up (SU) methods (both conventional and direct swim-up: CSU, DSU) [129,130,131]. In DGC, normal motile sperm cells are able to penetrate the higher densities in direction of the centrifugation force (the lower fraction), while immotile or abnormal sperm morphology are retained at the upper phase of the gradient or at the interphases [132,133]. One or two subsequent washing centrifugation steps allow to remove gradient medium [131], although it is recommended to minimize the number of centrifugations to avoid the production of ROS [134]. SU methods, instead, allow a selection of motile sperm by their ability to swim out of seminal plasma and into culture medium. The sample can be subjected to swim-up either with (CSU) or without (DSU) centrifugation [128,135]. Different comparative studies evaluated the effects of sperm preparation procedures on sperm quality in order to highlight which method was superior for isolating “functionally normal” sperm to be used for ART [131]. The literature is extremely inconsistent on this topic. Some studies focused their attention on the DGC method, observing the effect that this technique has on the semen sample as compared to the whole ejaculate. Some studies analyzed the generation of ROS and sperm DNA damage highlighting a positive correlation between centrifugal pelleting of unselected sperm or DGC and ROS production [134,136,137]. Conversely, other studies demonstrated an association between the DGC method and a higher mitochondrial membrane potential, lower DNA fragmentation and lower ROS production [138,139]. Similarly, some studies analyzed the effects that SU procedure may have on the semen quality. Henkel et al. reported a higher percentage of normal chromatin-condensed sperm after SU compared to the ejaculate [140]. In contrast, subsequent studies reported no correlation between SU and sperm DNA damage [141,142].

Controversial results were also obtained for studies comparing the effects of the two different techniques (DGC and SU) on sperm DNA integrity [131,143,144,145,146]. The group of Viswambharan and Murugan confirmed that both DGC and SU allow to obtain semen samples with lower DNA fragmentation compared to basal seminal fluid. In addition, they observed that DGC is more efficient than SU in isolating sperms with better DNA integrity [147]. Similarly, Sakkas and colleagues demonstrated the superiority of DGC on SU in terms of isolation of sperm with lower DNA damage [143,146]. These results were partially in contrast with those from other groups, who observed no significant differences between the two techniques [131,145,146]. In particular, Zini and colleagues demonstrated that the percentage of sperm with denaturated DNA is significantly lower in SU-treated but not in DGC-treated samples compared with the whole semen [144]. Muratori and colleagues evaluated the efficacy of both DGC and SU in removing DNA-damaged spermatozoa. Using a modified terminal deoxynucleotidyl transferase (TdT)-mediated fluorescein-dUTP nick end labeling (TUNEL) technique, they observed conflicting results: a fraction of subjects experienced an increase in DNA-damaged sperm during selection with DGC and SU, while a decrease in others [148]. These results are difficult to explain and require a more in-depth study of the seminal fluid biology and composition.

To further investigate this aspect, Ricci et al. proposed a new multiparameter flow cytometric method for semen analysis that includes sperm viability and apoptosis evaluation in order to compare the effect that the two different techniques have on sperm quality. They observed that both DGC and SU are efficient in reducing the percentage of apoptotic sperm compared to whole semen, suggesting that both these techniques help remove most of the apoptotic sperm. They also obtain a significantly lower percentage of apoptotic and necrotic sperm in SU-treated samples, but, at the same time, a significantly higher mean recovery rate of viable sperm after DGC compared to SU, suggesting that an ideal method does not exist [131].

The group of Yamanaka et al. analyzed semen samples from an ultrastructural point of view when the two techniques were used in combination. The ultrastructural abnormalities in sperm heads and tails were significantly lower compared to samples processed by DGC or SU alone. Moreover, the combination of DGC and SU was more effective in eliminating sperms with DNA fragmentation than DGC alone. Concluding, the authors claimed that the use of the DGC and SU in combination would be the best approach to use for the preparation of the semen sample, even if some ultrastructural abnormalities may remain [149].

Most of the studies mentioned in this section considered semen parameters such as the recovery rate, concentration, progressive motility rate, morphology and DNA fragmentation of the recovered sperm. There are still insufficient studies that specifically compare c-IVF outcomes after DGC or SU. For example, Van der Zwalmen and colleagues compared the pregnancy rate in c-IVF cycles after DGC or SU semen preparation methods, highlighting higher results by using the DGC treatment [150]. Overall, although the efficiency of DGC and SU has been compared since the 1980s, the results remain contradictory, but important differences in terms of isolation of “functionally normal” sperm do not seem to exist.

## 5. Conventional IVF and Fertilization Failure

### 5.1. The Role of the Rescue ICSI

The increase in the use of ICSI for non-male factors of infertility has been documented in the last decades as a key characteristic of ART cycles worldwide. As reported above, several reasons have been postulated to explain the increasing use of this technology, including a higher reimbursement associated with ICSI or an over-interpretation of possible advantages of ICSI such in case of a reduced number of oocytes, poor oocyte quality or advanced maternal age [5,151]. In this context, it is worth mentioning that reducing the chance of TFF represents another plausible reason for the preferential use of ICSI even in the absence of a male infertility factor. In fact, TFF is a disheartening result for both patients and professionals, with an incidence ranging between 5% and 20% after c-IVF [152]. The use of ICSI can reduce the risk of TFF even if evidence of improved fertilization results with ICSI is still debated and depends on the infertility indication [11,104,153,154]. According to the results of a recent systematic review, TFF risk significantly increases after c-IVF insemination compared to ICSI (relative risk = 2.63, 95%CI: 1.29–5.35) in couples with non-male-factor infertility [153].

Since TFF is an extremely negative experience, the possibility of identifying and recognizing possible risk factors is of particular interest. However, available data fail to a large extent to offer a valuable tool for predicting TFF due to prediction models with limited accuracy. In a previous review [155], a number of clinical prediction models for c-IVF developed in the last decades have been considered in order to estimate individualized chances of success; most of them include female age, duration of infertility, infertility factor, number of embryos transferred, number of previous cycles and embryo quality. However, even if a certain statistical power has been reached for predicting the cumulative chance of pregnancy under specific modeling requirements, a reliable tool for the prediction of fertilization rate in c-IVF or ICSI cycles is still lacking in the clinical practice. A recent large retrospective study with more than *n* = 100,000 c-IVF/ICSI cycles [156] investigated possible risk factors and proposed a prediction model for TFF; c-IVF was shown to be associated with a higher risk of TFF in presence of advanced female and male age, BMI between 24 and 28 kg/m^2^, low level of AMH and a reduced number of available oocytes. As a counterpart to the prediction strategy, efforts have been made to manage TFF when it occurs. In particular, the strategy of rescue ICSI (r-ICSI) has been proposed. This technique consists in performing ICSI as soon as oocytes show no signs of fertilization after c-IVF. Signs of fertilization such as the extrusion of the second polar body can be checked after 4–6 h after conventional in vitro fertilization or after 16–24 h when pronuclei are expected to appear; in the first condition, the rescue ICSI is usually indicated as “early r-ICSI”, while in the second condition as “late r-ICSI”.

A total of *n* = 1313 c-IVF cycles with TFF were reviewed with 2.933 r-ICSI embryos transferred in *n* = 1136 fresh and *n* = 71 frozen-thawed transfers [157]. The pregnancy rate for cases of r-ICSI after TFF was 15.6% per cycle. A subgroup analysis showed better results for early (4 studies) compared to late r-ICSI (27 studies): in cases of TFF, early r-ICSI resulted in a pregnancy rate of 44% compared to 10% obtained with late r-ICSI.

**Figure 1:**
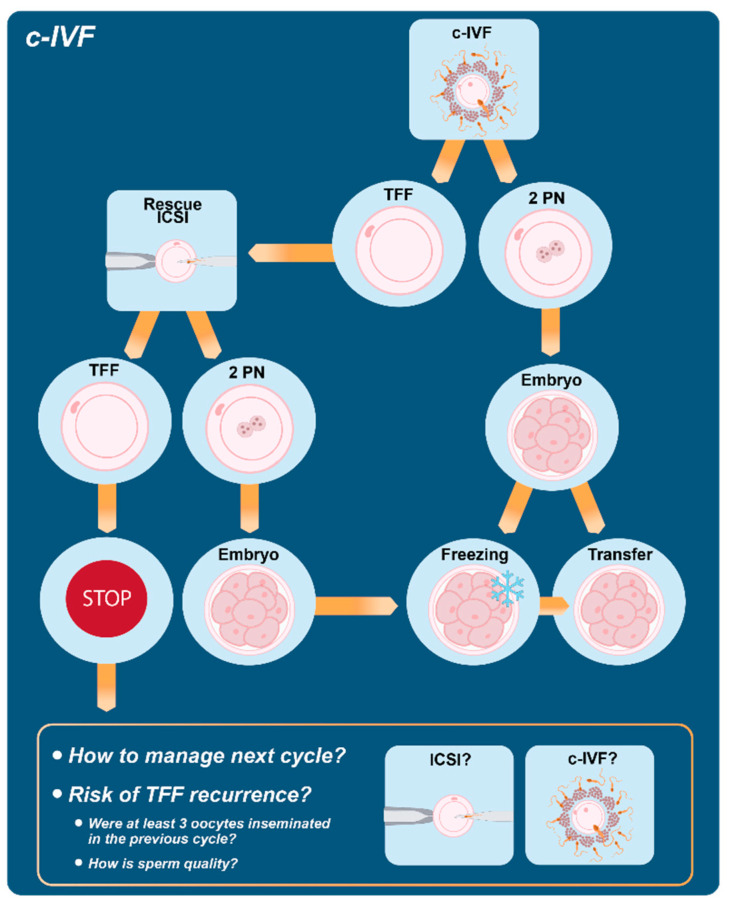
Flowchart describing possible hindrances and solutions related to c-IVF procedure.

Thus, r-ICSI has been proposed in order to potentially save cycles with total or partial fertilization failure even if its success rate is low, particularly in case of r-ICSI performed the day after c-IVF. Possible explanations of low rate of success, consistent with a better performance of early r-ICSI, may include time-dependent deterioration in oocyte competence and loss of synchronization between endometrial growth and embryo development. A breakthrough in the field of r-ICSI cycles has recently come from the observation that good results can be achieved when r-ICSI embryos are transferred after cryopreservation in a subsequent frozen-thawed cycle [158,159]. A recent review by our group [160] including 22 original studies on late r-ICSI confirmed unsatisfactory results in fresh cycles with clinical pregnancy rate per embryo transfer and implantation rate equal to 10% and 5%, respectively. On the contrary, transfer of cryopreserved r-ICSI embryos was shown to ameliorate success rates, with pregnancy rate per embryo transfer equal to 37% with an odds ratio (OR) = 4.7 (95% CI: 2.6–8.5). Similarly, a comparison of implantation rates between the transfer of supernumerary frozen r-ICSI embryos and fresh embryos showed an OR = 3.3 (95% CI: 2.0–5.5). From a safety point of view, available information does not suggest a significant increase in adverse outcomes following application of r-ICSI, including malformation rate, but it should be mentioned that less than 200 births have been described so far. According to the main results of the systematic review, the cryopreservation strategy could overcome most of the technical and biological issues associated with fresh transfer after late r-ICSI, thus possibly representing an interesting approach for couples experiencing TFF following c-IVF cycles. At the same time, this observation has important implications for clinical embryologists since it could increase the confidence in proceeding with c-IVF and promote its use while reducing the risk of TFF (Figure 1).

### 5.2. How to Manage the Next Cycle?

If a fertilization disorder is an unidentified cause of idiopathic infertility, then this may cause the recurrence of TFF in a subsequent ART cycle. It has been reported that the recurrence rate of TFF using c-IVF can be very high, up to 70% [161]. As a consequence, the majority of patients experiencing a TFF with c-IVF undergo a subsequent cycle with ICSI as a first choice for insemination. This strategy, coupled with a higher dose of gonadotropins aimed at retrieving a higher number of oocytes, is reasonably considered the best choice to limit the risk of TFF recurrence (Figure 1). In Krog et al. [162], *n* = 237 out of *n* = 304 (78%) TFF patients underwent a subsequent cycle, and 63% of those cycles were treated with ICSI achieving fertilization in 91% of cases; of note, fertilization was obtained also in a considerable proportion (76%) of TFF cases treated with c-IVF in the subsequent cycle. A similar figure was highlighted in the study by Lipitz et al. [163]. In order to shed more light on the comparative potential of c-IVF and ICSI, van der Westerlaken et al. [164] treated a small cohort of couples with c-IVF or ICSI on sibling oocytes after a first c-IVF attempt with no or low fertilization. They found a significantly higher fertilization rate in sibling oocytes treated with ICSI, with a recurrence rate of TFF in the c-IVF-treated oocytes ranging between 50% and 67%. The preferential use of ICSI in a subsequent cycle is in general more frequent among couples with a higher number of oocytes in the TFF cycle. This strategy is consistent with the observation that, when a reduced number of oocytes is available, the occurrence of TFF could be due to the sample size rather than to a biological limit. In fact, if we assume a fertilization rate per oocyte equal to 70%, at least three oocytes are needed in order to exclude that TFF is due to chance with a *p* value < 0.05 (probability of three independent events of fertilization failure with three oocytes = 30% × 30% × 30% = 2.7%). Besides fertilization rate, it would be of great benefit to clinicians to accurately counsel patients on their chance of pregnancy after a TFF cycle. Among TFF patients treated with a subsequent c-IVF, 20 out of 111 became pregnant in the report by Kinzer et al. [165]. The delivery rate was similar to that of patients in their second cycle without previous TFF or to TFF patients treated with ICSI in the subsequent cycle. Previous studies suggested that a reduced fertilization rate with c-IVF is a negative prognostic factor for successive ICSI cycles: Miller et al. [166] reported a 20% pregnancy rate compared to 47% of control ICSI. Similarly, Tomas et al. [167] found that in the group with previous failed/low fertilization rate, the pregnancy rate with ICSI was 20% compared to 34% of the group treated with ICSI for a male factor. The long-term reproductive prognosis up to 13 years was also reduced in TFF patients according to Krog et al. (2015): 50% (153/304) of the TFF patients succeeded in at least one live birth compared to 70% (212/304) [162]. In summary, reliable predictors of TFF recurrence after c-IVF are not available and ICSI is generally preferred in subsequent cycles even though the success rate is expected to be lower compared to patients without a history of fertilization failure, indicating that TFF patients as a group are more reproductively challenged. Indeed, the concept of TFF being explained by genetic incompatibility when it comes to altered variants expression of gamete fusion genes was recently brought up, suggesting that infertility as a term concerns the couple rather than a single partner [168].

## 6. Conclusions

We herein revised the current evidence for particular indications and underlying procedures associated with c-IVF. The overall impression is that this ART strategy remains a solid option that can be performed without rigid schemes, and that it must be avoided only in cases of severe male infertility or presence of antisperm antibodies and whose failure could presently be overcome. This is in line with the demonstration of a similar cumulative live birth rate when comparing ICSI with c-IVF for couples with non-male-factor infertility [8]. More specifically, evidence presented herein allowed us to challenge some popular beliefs on c-IVF such as its shortcomings in case of PGT, to raise awareness about alternative perspectives on specific controversial topics such as its use in presence of few eggs and to highlight the potential benefits of the r-ICSI (Table 5). We finally aim to introduce the concept of value, which is the balance between potential benefits, potential harms and cost of care [169]. Quantification of benefits and harms should be based on high-quality evidence, which is not always the case in the ART field. In many circumstances, the choice between ICSI and c-IVF is taken based on sub-optimal evidence or on evidence of coexistence of benefits and harms that must be balanced. In these conditions, the additional cost burden of ICSI, where data on improved live birth outcomes over conventional insemination are limited or absent, must be considered. In addition, safety issues are not thoroughly ascertained in the case of ICSI.

Keeping in mind that the goal of treatment is represented by a healthy baby and that TFF may only represent a surrogate outcome, scientific efforts should continue to assess the effectiveness, safety and clinical relevance of both c-IVF and ICSI.

## Figures and Tables

**Table 1:** Results deriving from the comparison between different incubation intervals of spermatozoa and oocytes in in vitro fertilization procedure.

Authors, Year	Interval of Co-Incubation	Fertilization Rate (FR) in Short IVF	Clinical Pregnancy Rate (CPR) in Short IVF	Implantation Rate (IP) in Short IVF
Gianaroli et al., 1996 [22]	1 h vs. overnight	Improved	Improved	Improved
Quinn et al., 1998 [29]	1 h vs. overnight	Unchanged	Improved	Improved
Coskun et al., 1998 [30]	1 h vs. overnight	Unchanged	Not assessed	Not assessed
Dirnfeld et al., 1999 [31]	1 h vs. overnight	Unchanged	Improved	Improved
Lin et al., 2000 [32]	1–3 h vs. overnight	Unchanged	Not assessed	Not assessed
Swenson et al., 2000 [33]	2 h vs. overnight	Not assessed	Worsened	Unchanged
Boone et al., 2001 [34]	3 h vs. overnight	Worsened	Not assessed	Not assessed
Lundqvist et al., 2001 [35]	2 h vs. overnight	Worsened	Unchanged	Unchanged
Dirnfeld et al., 2003 [21]	1 h vs. overnight	Unchanged	Not assessed	Not assessed
Kattera et al., 2003 [36]	2 h vs. overnight	Unchanged	Improved	Improved
Barraud-Lange et al., 2008 [37]	1 h vs. overnight	Worsened	Not assessed	Not assessed
Xiong et al., 2009 [26]	1–6 h vs. overnight	Unchanged	Unchanged	Not assessed
Dai et al., 2012 [38]	1–4 h vs. overnight	Unchanged	Unchanged	Unchanged
Huang et al., 2013 [39]	1–4 h vs. overnight	Not assessed	Improved	Not assessed
Zhang et al., 2013 [40]	1–6 h vs. overnight	Unchanged	Improved	Improved
Li et al., 2016 [19]	2 h vs. overnight	Unchanged	Improved	Improved
Le Bras et al., 2017 [27]	2 h vs. overnight	Worsened	Improved	Improved
He et al., 2018 [41]	4/6 h vs. overnight	Worsened	Unchanged	Unchanged
Chen et al., 2019 [23]	3/4 h vs. overnight	Unchanged	Unchanged	Unchanged
Kong et al., 2021 [24]	4 h vs. overnight	Unchanged	Unchanged	Not assessed

**Table 2:** Studies comparing c-IVF and ICSI outcomes in women with DOR in the absence of male factor infertility.

Author, Year	Design	Analysis Years	Number of Oocytes	Number of Patients/Cycles	Result
Moreno et al., 1998	Prospective	1996–1997	≤6, ≤3	IVF = 52 ICSI = 52	PR/IR/FR/FF/embryo quality = comparable
Luna et al., 2011	Retrospective	2002–2009	≤4	IVF = 179 ICSI = 171	CPR/FR/IR/FF/CR/MR = comparable
Tannus et al., 2017	Retrospective	2012–2015	≤3	IVF = 72 ICSI = 164	LBR/CPR/FF/FR = comparable
Liu et al., 2018	Retrospective	2011–2016	≤5	IVF = 534 ICSI = 110	CPR/LBR/MR/CR = comparable **IR = IVF 15.11% vs. ICSI 7.75%** **CLBR = IVF 14.59% vs. ICSI 5.56%** **FR = IVF 61.56% vs. ICSI 76.00%**
Guo et al., 2018	Retrospective	2012–2015	1, 2, 3 or 4	IVF = 870 ICSI = 435	CPR/CLBR = comparable
Drakopoulos et al., 2019	Retrospective Multicentre	2009–2014	1–3	IVF = 90 ICSI = 600	FR/LBR/CLBR = comparable
Supramaniam et al., 2020	Retrospective	1998–2016	≤3	IVF= 33,436 ICSI = 29,205	LBR/CPR/FF = comparable **FR = 2% lower for IVF**
Liu et al., 2020	Retrospective	2012–2016	≤6	IVF = 5071 ICSI = 734	LBR/all perinatal outcomes = comparable
Haas et al., 2020	Prospective	2018–2019	mean 4.3	IVF= 258 ICSI= 257	FR/number of cleavage-stage and top-quality embryo = comparable
Isikoglu et al., 2022	Retrospective	2017–2019	≤3	IVF = 77ICSI =65	IR/LBR/FF/CR = comparable **FR = IVF 85.68% vs. ICSI 72.58%**

Bold font in the results column denotes significant findings. CPR: clinical pregnancy rate; FR: fertilization rate; IR: implantation rate; FF: fertilization failure, CR: cancellation rate; MR: miscarriage rate; LBR: live birth rate; CLBR: cumulative live birth rate; PR: pregnancy rate.

**Table 3:** Findings of studies investigating outcomes in couples with ASA-positive male partners compared to ASA-negative male partners following c-IVF and ICSI.

Authors, Year	Design	Antibodies District	Fertilization Rate	Clinical Pregnancy Rate	Live Birth Rate
**c-IVF**					
Junk et al., 1986	Retrospective	Semen	**Reduced**	Not assessed	Not assessed
Acosta et al., 1994	Retrospective	Semen	**Reduced**	Reduced	Not assessed
Lähteenmäki et al., 1995	Retrospective	Semen	**Reduced**	Unchanged	Not assessed
Culligan et al., 1998	Retrospective	Semen	Unchanged	Not assessed	Not assessed
Vujisić et al., 2005	Prospective	Semen	Unchanged	Unchanged	Not assessed
Lu et al., 2019	Retrospective	Serum	**Reduced**	**Reduced**	**Reduced**
**ICSI**					
Nagy et al., 1995	Retrospective	Semen	**Increased**	Unchanged	Not assessed
Lähteenmäki et al., 1995	Retrospective	Semen	Unchanged	Unchanged	Not assessed
Lu et al., 2019	Retrospective	Serum	Unchanged	Unchanged	Unchanged

Bold font in the results column denotes significant findings.

**Table 4:** Results deriving from the comparison of conventional IVF vs. ICSI for PGT-M, PGT-A and FISH analyses.

Authors, Years	Design	Analysis Years	Insemination Technique	Fertilization Rate (%)	Embryos Analyzed (%)	Euploid Embryos (%)	*p*
**PGT-M**							
Feldman et al., 2017	Cohort-historical	2006–2014	c-IVF	696%	84.2%	38.9%	n.s.
			ICSI	58.8%	86.3%	36.2%	n.s.
**PGT-A**							
Palmerola et al., 2019	Retrospective	2015–2017	c-IVF	61.8%	25.7%	27.9%	n.s.
			ICSI	61.4%	74.3%	30.0%	n.s.
De Munck et al., 2020	Single-center prospective	2018–2019	c-IVF	64.0%	67.4%	49.8%	n.s.
			ICSI	65.4%	60.6%	44.1%	n.s.
**Authors, years**			**Insemination techniques**	**No. of embryos analyzed**		**Aneuploid embryos (%)**	***p***
**FISH**							
Sahin et al., 2017	Retrospective	NR	c-IVF	57		65.0%	n.s.
			ICSI	183		69.9%	n.s.

NR = not reported.

**Table 5:** c-IVF versus ICSI in the absence of a male factor of infertility: summary of main findings.

Indication	Main Findings
Advanced maternal age	Most available data fail to demonstrate an advantage of ICSI over c-IVF in terms of fertilization rate, embryo development rate, pregnancy and live birth rates according to the insemination technique.
Decreased ovarian reserve	Fertilization rate, fertilization failure, implantation rate, clinical pregnancy rate and live birth rate are comparable after c-IVF and ICSI.
Endometriosis	A higher fertilization rate is reported using ICSI, without a significant advantage in terms of implantation rate, pregnancy rate, chemical pregnancy, clinical abortion and ongoing pregnancy rate compared to c-IVF.
Autoimmunity	Lower fertilization, clinical pregnancy and live birth rates are documented in partners of antisperm antibodies positive men treated with c-IVF. ICSI can overcome these issues. Superiority of ICSI over c-IVF in couples with thyroid autoimmunity has not been documented.
Preimplantation genetic testing	Comparable percentages of embryos with a complete diagnosis and comparable percentages of unaffected/transferable embryos are obtained with c-IVF and ICSI in cycles with genetic testing for aneuploidy. No significant differences in contamination rates of the washing medium samples after c-IVF or ICSI are reported.
Single oocyte retrievals	Fertilization, implantation and live birth rates per oocyte retrieval are comparable using c-IVF or ICSI.

## Data Availability

Not applicable.
